# Transcriptional signatures of rapid protection from Sudan virus infection by a single dose of a vesicular stomatitis virus-based vaccine

**DOI:** 10.1371/journal.ppat.1014143

**Published:** 2026-04-10

**Authors:** Brianna M. Doratt, Sheridan B. Wagner, Delphine C. Malherbe, Geoffrey Smith, Andrea Marzi, Ilhem Messaoudi

**Affiliations:** 1 Department of Microbiology, Immunology, and Molecular Genetics, College of Medicine, University of Kentucky, Lexington, Kentucky, United States of America; 2 Laboratory of Virology, Division of Intramural Research, National Institute of Allergy and Infectious Diseases, National Institutes of Health, Hamilton, Montana, United States of America; NIAID: National Institute of Allergy and Infectious Diseases, UNITED STATES OF AMERICA

## Abstract

Sudan virus (SUDV) has caused multiple outbreaks of human disease with case fatality rates ranging from 41 to 100%. We have previously shown that a single vaccination with a recombinant vesicular stomatitis virus-based vaccine expressing the SUDV-Gulu glycoprotein (VSV-SUDV) prevented clinical and fatal disease from lethal SUDV challenge in cynomolgus macaques. With the high probability of future outbreaks, it is critical to determine the molecular mechanisms of VSV-SUDV-mediated protection and the ability to impart rapid protection against SUDV infection. In this study, RNA from whole blood samples obtained from nine cynomolgus macaques that were challenged with SUDV-Gulu post-vaccination with either VSV-EBOV (28 days before challenge, n = 3) or VSV-SUDV (28 or 7 days before challenge, n = 3/group) was subjected to bulk RNA sequencing. EdgeR, STEM, MaSigPro, and CIBERSORTx were used to assess longitudinal transcriptional changes elicited by vaccination and challenge. Our analysis revealed that VSV-SUDV and VSV-EBOV elicited distinct transcriptional responses. Moreover, NHPs vaccinated with VSV-EBOV (non-protective) generated a transcriptional response following SUDV challenge indicative of dysregulated inflammation. In contrast, NHPs that received VSV-SUDV vaccine generated a transcriptional response indicative of a recall adaptive immune response. Finally, *in-silico* deconvolution methods indicated changes in immune cell frequency consistent with immune response and resolution in the VSV-SUDV-vaccinated NHPs that are not observed with VSV-EBOV-vaccinated NHPs. These data indicate that VSV-SUDV vaccination results in a protective humoral response as late as 7 days before challenge despite transcriptional evidence of subclinical features of infection.

## Author summary

Sudan virus (SUDV) is a highly fatal human pathogen with no approved vaccine. While several prior studies evaluated the transcriptional response to vaccination with vesicular stomatitis virus (VSV) based vaccines targeting different filovirus species, none have examined the transcriptional response to VSV-SUDV. Using a nonhuman primate model, we investigated the molecular responses following vaccination and subsequent lethal challenge. Gene expression analysis revealed that vaccination with VSV-SUDV elicited a robust immune response, distinct from that induced by a related Ebola virus vaccine, which did not confer protection. Importantly, the protective response was evident even when vaccination occurred as late as seven days prior to viral challenge. These findings enhance our understanding of the mechanisms underlying vaccine-induced protection against Sudan virus and support the potential use of VSV-SUDV as a rapid-response vaccine to contain future outbreaks.

## Introduction

Sudan virus (SUDV), a member of the *Filoviridae* family [1], was first identified in 1976 [2] and is the causative agent of Sudan virus disease (SVD) which has a case fatality rate ranging from 41 to 100% [3]. Despite the potential impact of SVD outbreaks on global public health, no efficacious vaccine is currently approved. Several live-attenuated recombinant vesicular stomatitis virus- (rVSV) or adenovirus-based vaccines engineered to target other closely related members of the *Filoviridae* family have shown limited to no cross-protection against SUDV exposure in animal models [4–6]. The lack of cross-protection is not surprising considering there is only a 57·8% sequence homology [7] of the viral glycoproteins. Most vectored filovirus vaccines are designed to include the full-length glycoprotein gene (GP) which encodes the precursor glycoprotein that is cleaved to express the surface and transmembrane subunits (GP1 and GP2) which together form the GP1,2 protein.

This lack of heterologous protection emphasizes the need for species-specific vaccines against pathogenic filoviruses [8]. Multiple vaccines targeting SUDV have been generated [9] including a bivalent adenovirus-vectored vaccine expressing both SUDV and EBOV GP (ChAdOx1-biEBOV) that failed to protect cynomolgus macaques from fatal SUDV disease [10]. Two recombinant subunit protein vaccines including a monovalent, SUDV GP only, and a bivalent, SUDV with Marburg virus (MARV) GP protected against lethal SUDV challenge in a three dose regimen [11]. Additionally, ChAd3-SUDV, a chimpanzee adenovirus expressing SUDV GP, and a recombinant VSV in which the G protein was replaced with SUDV-Gulu GP - rVSV∆G-SUDV-GP- (VSV-SUDV), provided protection following a single dose [12,13]. We showed that a single VSV-SUDV vaccination prevented clinical and fatal disease in cynomolgus macaques when administered 7 days before homologous viral challenge, similar to the rapid protection by VSV-vectored vaccines against other filoviruses [12,14–16]. However, the molecular underpinnings of VSV-SUDV-mediated protection have yet to be elucidated.

Here, we expanded upon our previous work and interrogated the transcriptional changes induced by VSV-SUDV when administered 28 or 7 days before challenge. A control group vaccinated with VSV-EBOV was not protected from SUDV challenge. VSV-SUDV induced a distinct transcriptional response to vaccination compared to VSV-EBOV. The transcriptional response following SUDV challenge in VSV-SUDV-vaccinated NHPs showed a recall immune response compared to a dysregulated response in VSV-EBOV vaccinated NHPs. Deconvolution methods indicated immune cell frequency fluctuations consistent with immune response and resolution with VSV-SUDV not observed with fatal disease.

## Results

### VSV-SUDV vaccination elicits a distinct transcriptional response compared to VSV-EBOV vaccination

**Fig 1:**
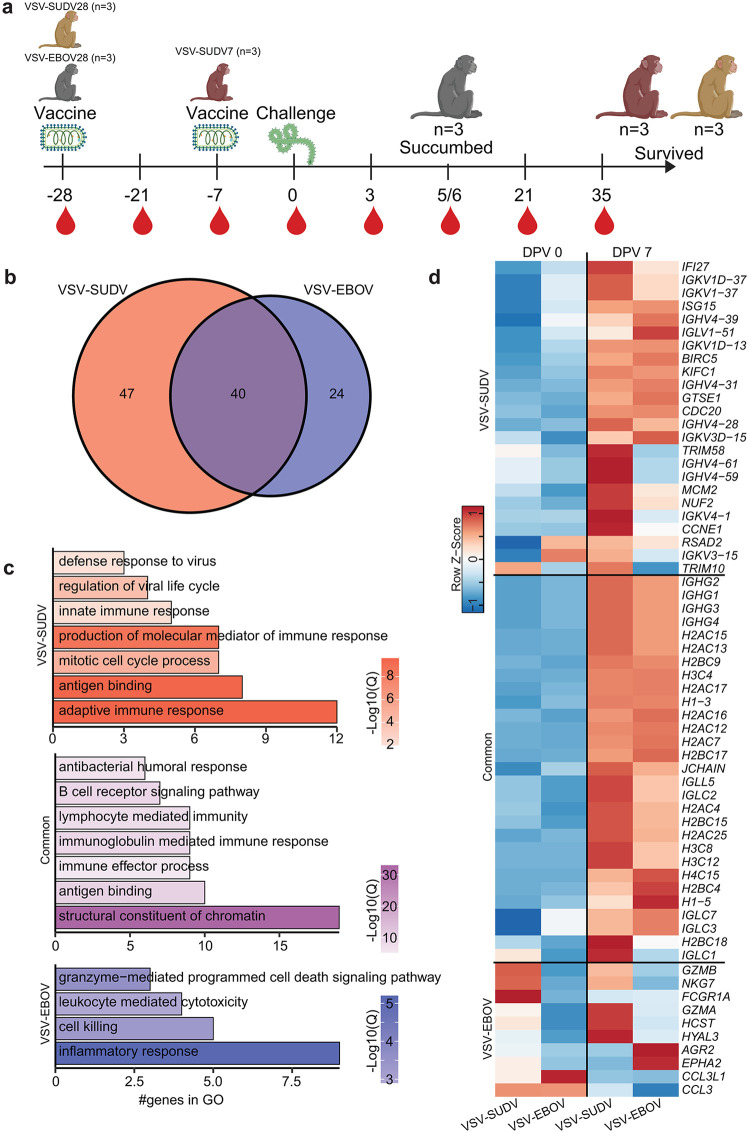
Transcriptional alterations following vaccination. **(A)** Experimental design. Three groups of n = 3 cynomolgus macaques were vaccinated at days post challenge (DPC) -7 with VSV-SUDV (VSV-SUDV7), DPC-28 with VSV-SUDV (VSV-SUDV28), or DPC-28 with VSV-EBOV (VSV-EBOV28). Animals were challenged with SUDV at DPC0. RNA was extracted from whole blood samples at the indicated timepoints and subjected to bulk RNAseq. Created in BioRender. Doratt, B. (2026) https://BioRender.com/9rrcx8e **(B)** Venn diagram of DEGs between DPV 7 relative to DPV 0 for the VSV-SUDV (grouping VSV-SUDV7 and VSV-SUDV28) and VSV-EBOV28 groups. **(C)** Bar graph representing GO terms for DEGs. Color indicates log10(q.value). Length indicates the number of genes. **(D)** Heatmap of average transcripts per million (TPM) for selected DEGs.

Longitudinal transcriptional signatures in whole blood samples after vaccination with a VSV vaccine expressing the SUDV-Gulu GP (VSV-SUDV, n = 6) were compared to those detected in control animals vaccinated with VSV vaccine expressing EBOV-Kikwit GP (VSV-EBOV, n = 3) (Fig 1A). Overall, vaccination with VSV-SUDV generated more DEGs 7 days post vaccination (DPV) compared to VSV-EBOV, indicating a larger early transcriptional response. The 40 shared DEGs mapped to chromatin modifications (*H1-5, H2AC16*) and B cell effector/humoral responses (*JCHAIN, IGHG2*) (Fig 1B–1D and S1 Table). The 47 DEGs unique to VSV-SUDV mapped to innate responses to virus (*ISG15, IFI27, TRIM10)*, cell cycle (*CCNE1, CDC20, KIFC1)*, and humoral immunity (*IGHV4–31, IGHV4–28*) (Fig 1B–1D and S1 table). The 24 DEGs unique to VSV-EBOV indicated upregulation of granzyme mediated cell killing (*GZMA*, *GZMB, NKG7*) and downregulation of chemotaxis (*CCL3, CCL3L1*) (Fig 1B–1D and S1 Table).

### Transcriptional response to SUDV challenge indicates a controlled inflammatory response in VSV-SUDV vaccinated NHPs

While prior work has demonstrated the efficacy of VSV-SUDV against fatal disease when administered 28 days before challenge in NHPs with and without pre-existing immunity to EBOV [6,12], no studies to date have characterized the transcriptional responses associated with success or failure. Therefore, we used Short Time-series Expression miner (STEM) to define clusters of genes with similar longitudinal transcriptional changes post-challenge with SUDV [17]. VSV-EBOV28 animals succumbed to lethal SUDV challenge at 5DPC (Fig 1A) and had two unique gene clusters with significant longitudinal changes. Gene expression in cluster_1 (1,842 genes) decreased by half between 0DPC and 3DPC before rebounding to slightly above initial levels at 5DPC (S1A Fig). Genes in cluster_1 enriched to processes associated with “T cell activation” (*CD3E, IL2RG*), “myeloid cell differentiation”, “mitotic cell cycle” (*CCND2, CDK11B, KIF2A*), and “hemopoiesis” (*ACTN1, DOCK2*) (S1B and S1D Fig and S1 Table). The expression of the 1,439 genes in cluster_2 increased by 4·7-fold at 5DPC (S1A Fig). Those genes were primarily involved in antiviral and inflammatory processes notably mapping to gene ontology (GO) terms “response to cytokine stimulus” (*IRAK2, MYD88, IFI27*), “viral process” (*CD81, CD55, APOBEC3C*), “response to type 1 and 2 interferons” (*STAT1/2, TLR2, IFITM1*), and “innate immune responses” (*CD14, IRF5, LYN, C1QC, RIGI)* (S1C and S1E Fig and S1 Table).

**Fig 2:**
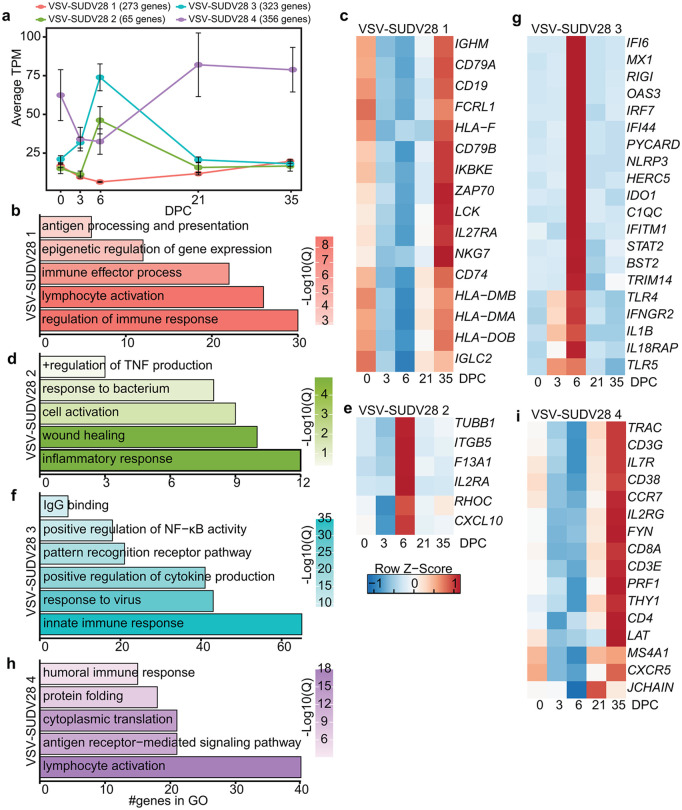
Transcriptional alterations associated with protection from lethal disease. **(A)** Line graph of the average TPM values for each gene cluster post challenge for VSV-SUDV28. (B,D,F,H) Bar graph representing GO terms for DEGs. Color indicates log10(q.value). Length indicates the number of genes. (C,E,G,I) Heatmap of average TPM values of selected DEGs.

VSV-SUDV vaccination was protective against SUDV challenge allowing us to profile gene expression up to 35DPC (Fig 1A). Four gene clusters were identified using STEM (Fig 2A). VSV-SUDV28 cluster_1 was composed of 273 genes, expression of which decreased by 30% at DPC6 following challenge before returning to baseline at 35DPC (Fig 2A). Cluster_1 genes play a role in “regulation of the immune response” (*CD14, CD19, FOXP1*), “epigenetic regulation of gene expression” (*KMT2A, H3C4, H3C8*), and “antigen processing and presentation” (*CD1C, CD74, HLA-DMA*) (Fig 2B and 2C and S1 Table). The expression of the 65 genes in cluster_2 peaked at 5/6DPC with a 3-fold increase before returning to baseline at 21DPC (Fig 2A). Cluster_2 genes mapped primarily to inflammatory processes such as “inflammatory response” (*IL2RA, CXCL10*) and “wound healing” (*RHOC, F13A1, ITGB5*) (Fig 2D and 2E and S1 Table). Expression of 323 genes in cluster_3 also peaked at 6DPC (3·5-fold increase) before returning to baseline at 21DPC (Fig 2A). Cluster_3 genes mapped to processes associated with “innate immune response” (*CD1D, IFI6, TLR4*), “response to virus” (*MAPK14, IFI6, STAT2, IRAK3*), as well as “IgG binding” (*FCER1G*) (Fig 2F and 2G and S1 Table). Finally, cluster_4 is composed of 356 genes that were decreased by 50% at 3–6DPC before rebounding at 21DPC (Fig 2A). Interestingly, cluster_4 genes were primarily associated with adaptive immune responses; “lymphocyte activation” (*CD3E/G, MS4A1*), “antigen receptor-mediated signaling pathway” (*TRAC, CD38*), and antibody production including “protein folding” and “humoral immune response” (*MS4A1, JCHAIN, IGHA1*) (Fig 2H and 2I and S1 Table).

### VSV-SUDV Vaccination close to challenge leads to sustained humoral immunity

**Fig 3:**
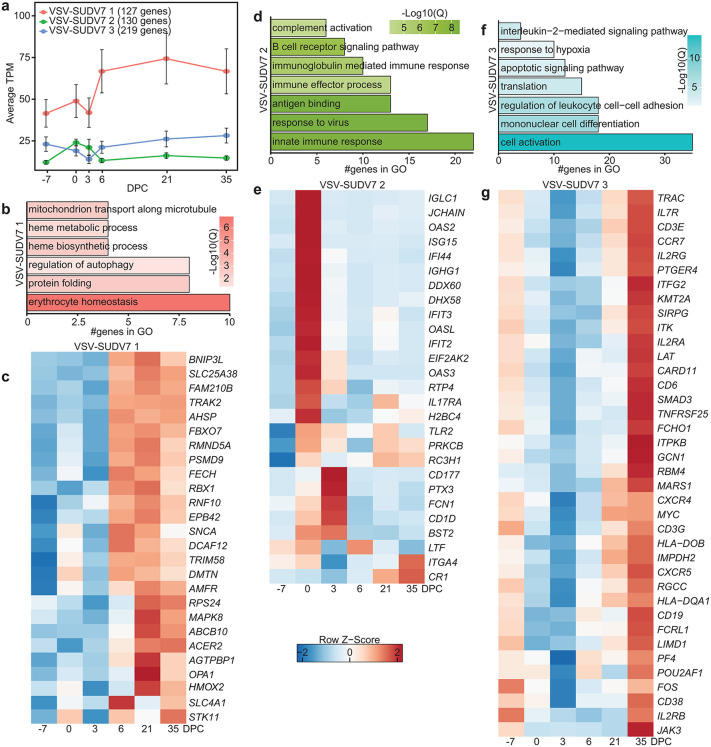
Transcriptional alterations correlating to timing of vaccination. **(A)** Line graph of the average TPM values for each gene cluster for the VSV-SUDV7 group. (B,D,F) Bar graph representing GO terms for DEGs. Color indicates log10(q.value). Length indicates the number of genes. (C,E,G) Heatmap of average TPM values of selected DEGs.

The protection afforded by vaccination 7 days before challenge (VSV-SUDV7, n = 3) enabled characterization of the transcriptional response up to 35DPC (Fig 1A). STEM identified 3 different gene clusters with distinct expression kinetics throughout the vaccination and challenge phases (Fig 3A). The expression of VSV-SUDV7 cluster_1 (127 genes) increased after vaccination and further increased after challenge (1·7-fold) through 35DPC (Fig 3A). These genes mapped to erythrocyte homeostasis (*EPB42, SLC4A1, FAM210B*), and heme metabolism/biosynthesis (*HMOX2*, *FECH, ABCB10, SLC25A39*) (Fig 3B and 3C and S1 Table). Genes in cluster_2 (130 genes) were upregulated 2-fold immediately after vaccination and returned to baseline by 6DPC (Fig 3A). These genes enriched to “innate immune response” (*IFI44, TLR2, ISG15*) and “immunoglobulin mediated immune response” (*IGHG1, JCHAIN, IGLC1*) (Fig 3D and 3E and S1 Table). Cluster_3 consisted of 219 genes downregulated 0·6-fold following vaccination through 3DPC before returning to baseline (Fig 3A). They enriched to “cell activation” (*CXCR5, IL2RA*) and “leukocyte cell-cell adhesion” (*SIRPG, SELP*) (Fig 3F and 3G and S1 Table).

### Rapid protection against lethal SUDV infection is linked to early and sustained upregulation of key metabolic genes

**Fig 4:**
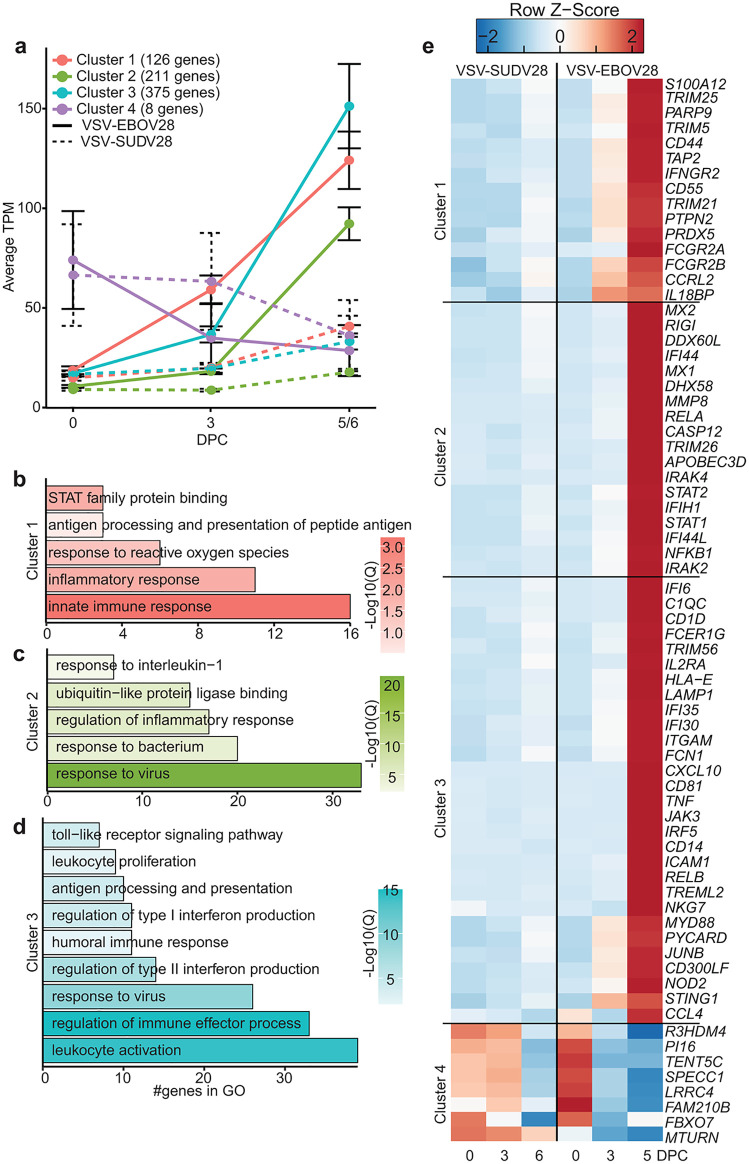
Comparison of transcriptional responses with protective vaccination. **(A)** Line graph of the average TPM values for each gene cluster for VSV-EBOV28 (solid line) and VSV-SUDV28 (dashed line). **(B-D)** Bar graph representing GO terms for DEGs. Color indicates log10(q.value). Length indicates the number of DEGs. **(E)** Heatmap of average TPM values of selected DEGs.

We next used the Microarray Significant Profiles (MaSigPro) to identify clusters of gene whose expression profile is different between two conditions [18]. We first compared the transcriptional responses following VSV-SUDV28 and VSV-EBOV28 vaccinations to define gene clusters that differentiate survival and fatal outcomes. We identified 4 clusters of genes with significant differences in longitudinal expression (Fig 4A). Clusters 1–3 (126, 211, and 375 genes respectively) were upregulated in both groups but to a higher magnitude in the VSV-EBOV28 group. Cluster 4 was downregulated earlier in VSV-EBOV28 (3DPC, 0·47 fold) compared to VSV-SUDV28 (6DPC, 0·54-fold) (Fig 4A). Clusters 1–3 mapped to GO terms associated with innate immune processes like “innate immune responses” (*C1QC, PYCARD, RIGI*) and “toll-like receptor signaling pathway” (*CD55, IRAK2, TRIM5, MYD88*), “antigen processing and presentation” (*TAP2, HLA-E*); antiviral immunity including “regulation of type I interferon production” (*RIGI*, *STAT1, IFI44)*; and, effector immune functions including “leukocyte activation” and “humoral immune response” (Fig 4B–4E and S1 Table). Cluster 4 included 8 genes involved in cell cycle regulation (*FBXO7, SPECC1, R3HDM4*) and cell signaling (*MTURN, PI16, FAM210B*) (Fig 4E and S1 Table).

**Fig 5:**
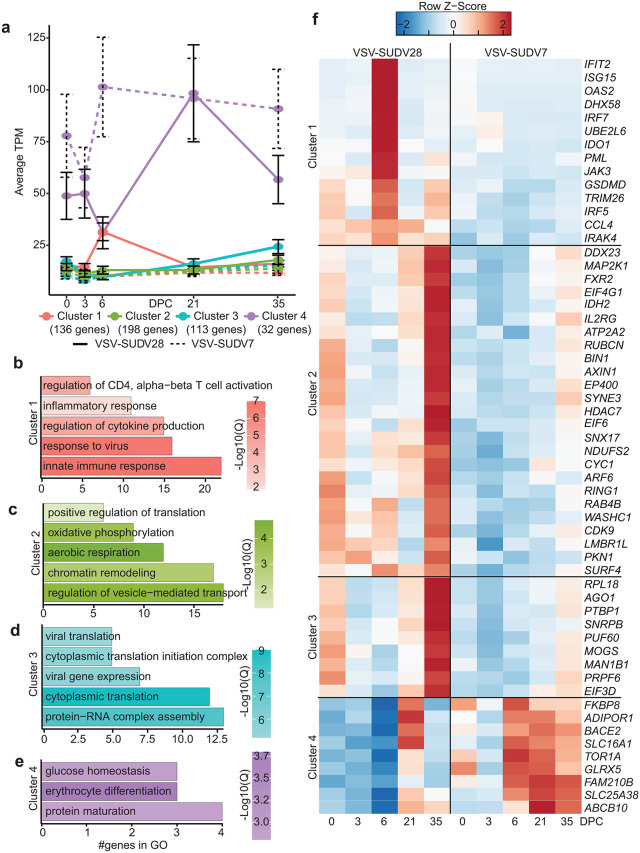
Impact of vaccine timing on transcriptional responses. **(A)** Line graph of the average TPM values for each gene cluster for VSV-SUDV28 (solid line) and VSV-SUDV7 (dashed line). **(B-E)** Bar graph representing GO terms for DEGs. Color indicates log10(q.value). Length indicates the number of DEGs. **(F)** Heatmap of average TPM values of selected DEGs.

We used MaSigPro to compare transcriptional responses of VSV-SUDV7 to VSV-SUDV28 post-challenge to identify the impact of vaccine timing on post-challenge responses. Cluster 1 (136 genes) was upregulated 2·1-fold at 6DPC in VSV-SUDV28 only (Fig 5A). These genes mapped to “innate immune response” (*IRF5, ISG15*), “response to virus” (*IFIT2, CCL4, OAS2*), and “regulation of cytokines” (*ISG15, IRF7, JAK3*) (Fig 5B and 5F and S1 Table). Expression of the genes in clusters 2 (198 genes) and 3 (113 genes) increased by 1·24- and 1·41-fold, respectively, at 35DPC in the VSV-SUDV28 group (Fig 5A) and mapped to “chromatin remodeling” (*HDAC7, RING1, CDK9*), metabolism (“aerobic respiration” and “oxidative phosphorylation”; *ATP2A2, CYC1, NDUFS2*), and “cytoplasmic translation” (*RPL18, EIF3D*) (Fig 5C, 5D and 5F and S1 Table). Cluster 4 consisted of 32 genes whose expression in the VSV-SUDV7 group increased at 6DPC (1·4 fold) and was sustained though 35DPC but initially decreased in VSV-SUDV28 group at 6DPC (0·6 fold) before a 2-fold increase at 21DPC and return to baseline at 35DPC (Fig 5A). These genes play a role in “erythrocyte differentiation” (*ABCB10, FAM210B*), “protein maturation” (*SLC16A1, BACE2, ADIPOR1*), and “glucose homeostasis” (*GLRX5, TOR1A, BACE2*) (Fig 5E and 5F and S1 Table).

### Altered cell frequencies with SUDV challenge demonstrate a recall adaptive immune response dependent on vaccine timing

To further characterize the immune landscape following SUDV challenge, we used CIBERSORTx to provide in silico estimates of cell type frequencies from transcriptional data of the collected whole blood samples [19,20]. In the VSV-EBOV28 group, our analysis revealed transcriptional changes indicative of an increase in the frequency of monocytes and regulatory T cells (Tregs) following challenge. Conversely, there was a decrease in memory B cells, memory CD4 T cells, and natural killer (NK) cells (S2 Fig). For the VSV-SUDV28 group, we observed a significant decrease in NK cells and naïve CD4 T cells as early as 3DPC, with memory CD4 T cells beginning to decrease at 5/6DPC before expanding at 21DPC. Monocyte and Treg frequencies increased early after challenge (3DPC). Interestingly, all cell types rebounded to near baseline with the notable exception of a significant increase in CD8 T cell frequencies beginning at 21DPC and maintained through 35DPC (S2 Fig). In the VSV-SUDV7 group, memory B cells increased as early as 5/6DPC while NK cells and naïve CD4 T cell frequencies increased only at 35DPC (S2 Fig).

## Discussion

Due to the significant threat that filovirus disease outbreaks pose to public health, the development of efficacious vaccines that provide rapid protection against these viruses is needed. Many vaccine platforms are being investigated to fill this critical gap including those based on viral vectors (VSV, Adenovirus), protein subunits, virus-like particles, DNA and mRNA. Many of these platforms are limited in their utility due to factors complicating deployment in an outbreak setting including the need for a multi dose regimen [9]. VSV vectored vaccines have been shown to be effective with single low dose vaccinations providing rapid complete protection from fatal disease for multiple viruses including SUDV, EBOV, and MARV as little as 7 days before challenge, with VSV-EBOV and VSV-MARV providing partial protection as fast as 3 days before challenge [12,14,15].

We observed the development of innate and adaptive immune responses to vaccination in the VSV-SUDV and VSV-EBOV groups at 7DPV as described for other VSV-vectored filovirus vaccines [16,21]. Both vaccines induced a strong B cell response in agreement with the critical and sufficient role of humoral responses to mediate VSV-based protection against filovirus infection [22,23]. Interestingly, the response to vaccination was more robust in the VSV-SUDV group with nearly double the number of DEGs. This observation is in line with our prior findings that VSV-SUDV elicited a more robust humoral response including antibody-dependent cellular phagocytosis (ADCP) and antibody-dependent complement deposition (ADCD) even when administered as little as 7 days before challenge compared to VSV-EBOV [12]. The observed ADCP and ADCD development was earlier and more robust with administration of the vaccine at 28 days before challenge [12]. Both viruses utilize the same receptor (NPC1) and cellular attachment factors (TIM-1 and C-Type lectins) [24] but SUDV GP1,2 has a 9-times higher affinity for NPC1 compared to EBOV-Mayinga GP1,2 due to changes in four key residues [25] which are conserved between EBOV-Mayinga and EBOV-Kikwit. Additionally, while these VSV based vaccines are attenuated they remain replication competent. Whether the enhanced binding to the cellular receptor results in increased replication or broader tissue tropism for VSV-SUDV *in vivo* could be investigated in future studies. However, the number of VSV RNA copies was equivalent in all groups at all measured times post vaccination [12]. This suggests that the enhanced NPC1 binding affinity of SUDV GP1,2 could potentially influence the magnitude and quality of immune responses elicited by vaccination with this vector and lower the activation threshold needed to respond to during challenge.

Both EBOV and SUDV cause severe hemorrhagic disease with development of granulocytosis, lymphopenia, and thrombocytopenia. Lethal EBOV infection is known to provoke a rapid and sustained cytokine storm, with elevation of several pro-inflammatory cytokines including TNFα, IFN, and IL-6, which closely correlate with disease severity and fatality rates [26,27]. Similarly, SUDV infection was associated with dramatic upregulation of several pro-inflammatory cytokines including IP-10, IL-6, and MCP-1 [28]. Post SUDV challenge, we observed large transcriptional changes in the VSV-EBOV28 (unprotected) group suggestive of uncontrolled immune activation in line with a cytokine storm [12]. Indeed, VSV-EBOV, was non-protective against lethal SUDV challenge with these animals developing viremia, large dysregulation in cytokine profiles and gross pathological changes in multiple organs, while all animals vaccinated with VSV-SUDV showed little to no clinical signs of disease [12].

Our *in silico* analysis showed a loss of memory B cells and memory activated CD4 T cells, as observed in individuals infected with filoviruses [28–30]. Indeed, we have previously shown that these same animals vaccinated with VSV-SUDV had no change in frequency of white blood cells while those vaccinated with VSV-EBOV developed lymphopenia starting on 3DPC [12]. This decrease in memory cells may be a consequence of the dysregulation and impairment of the innate immune response after filovirus infection [31]. Additionally, the phospholipid phosphatidylserine (PS), which is present on the viral envelope, has been shown to uniquely bind to the Tim-1 cellular attachment factor on host cells [32]. In EBOV infection, PS preferentially interacts with Tim-1 + memory CD4 T cells and causes abortive infection and death of these non-permissive cells [29,30,33]. While no study to date has been performed on the interaction of PS and Tim-1 + B cell in filovirus infections, it has been shown that specific subsets of memory and regulatory B cells express significant levels of Tim-1 [34,35]. Therefore, it could be hypothesized that SUDV could also selectively deplete memory cells via abortive infections triggered by interaction of PS and Tim-1. Despite observed lymphopenia and a subsequent loss of adaptive memory population, we observed upregulation of the expression of key adaptive immune cell markers such as *CD3* and *CD19*. Signaling in T cells via Tim-1 requires binding to CD3 and a subsequent association with the TCR complex [36]. Additionally, CD3 is more highly expressed on naïve T cells, the remaining subset after the selective depletion of memory subsets via abortive infection [37].

Additionally, our transcriptional analysis revealed an upregulation of genes associated with immune activation (*NF-kB*), complement (*C1QC*) and myeloid cells (*CD14*). These pathways are key components of an innate immune response to infection. Activation of the complement pathways has been demonstrated in filovirus vaccination and infections where it plays a protective role via ADCD, which was significantly increased in VSV-SUDV vaccinated animals [12,38,39]. Complement activation could have a detrimental role in filovirus infection with the development of antibody-dependent enhancement (ADE); although demonstrated in vitro with EBOV-specific monoclonal antibodies, ADE was not observed in this cohort [12,38,39]. This dichotomy in complement-mediated responses may also be observed in our data where *C1QC* is upregulated in both the VSV-EBOV28 and VSV-SUDV28 group at 5/6 DPC with differing survival outcomes. NF-kB is central to viral infection responses and is a key regulator of monocyte activation response, including in the production of inflammatory cytokines potentially contributing to the development of a cytokine storm [40,41]. During infection or inflammation, complement activation and NF-κB signaling jointly promote monocyte recruitment and activation [40,42], a hallmark of filovirus infection [28]. Indeed, our *in silico* analysis demonstrated a rapid upregulation in the predicted frequencies of monocytes. Monocytes are a major driver of fatal disease progression as they are highly permissive to filovirus infection, become rapidly activated and produce large quantities of pro-inflammatory cytokines aligning with the sustained monocytosis observed in the VSV-EBOV28 group which succumbed to infection [43,44]. We have previously shown that vaccination with VSV-EBOV against SUDV produced a large cytokine storm that was not observed with VSV-SUDV vaccination [12].

The transcriptome of the VSV-SUDV28 group showed multiple distinct dynamic shifts, indicating that this vaccine schedule allows the immune system to complete the immune maturation process, leading to a robust and dynamic recall response upon challenge, as characterized in many vaccination studies [45,46]. Additionally, while the transcriptional changes within the VSV-EBOV28 group suggested lymphopenia, the initial decrease in memory activated CD4 T cells at DPC6 in the VSV-SUDV28 group is likely a reflection of lymphocyte migration into tissues [47,48]. Interestingly, we also observed a concurrent upregulation in the expression of IL-2 receptor genes *IL2RA* (5/6DPC) and *IL2RG* (21DPC). IL-2 and its receptors are essential for the proliferation and survival of both CD4+ and CD8 + T cells following antigen stimulation, where IL-2 first binds to the low affinity membrane bound IL2RA subunit, then recruits IL2RB and IL2RG which have cytoplasmic components, forming the high affinity signaling complex [49,50]. Following the upregulation of IL-2 receptors we observed an expansion of both the memory activated CD4 and CD8 T cells at DPC21. Enhanced IL-2 signaling after vaccination and challenge increases the number and effectiveness of antigen-specific T cells [51]. These changes in T cell subset frequency and elevated IL-2 receptor expression, are indicative of effective vaccine-induced cellular immunity not observed in other groups. Finally, monocytosis in the VSV-SUDV28 was less significant than the one observed in the VSV-EBOV28 group and resolved to baseline quickly. This change may indicate reactivation of the innate immune response that limits viral replication and infection and activation of monocytes [44] especially as this cohort experienced transient viremia at 6DPC [12]. This dynamic response has been shown functionally with longer interval filovirus vaccinations resulting in stronger neutralizing antibody response, ADCP, and shift in T cell memory responses including in this cohort [12,14]. Altogether these data reveal that the VSV-SUDV administration 28 days before challenge leads to a robust recall response and immune memory.

The interval between administration of VSV-based filovirus vaccines and subsequent challenge with filoviruses is a critical determinant of vaccine efficacy and immune response quality, with complete protection typically conferred with vaccination -7DPC and partial protection achievable as late as -3DPC [6,14,15]. Similarly, VSV-SUDV confers complete protection from clinical SUDV disease when administered as late as -7DPC in this cohort of animals [12]. The VSV-SUDV7 group exhibited fewer temporal shifts in gene expression following vaccination and during the early period after viral challenge compared to the VSV-SUDV28 group. For VSV-SUDV7, these patterns stabilized early at 6DPC and remained consistent through 35DPC. Similar patterns have been observed with VSV-MARV and VSV-EBOV vaccination administered shortly before challenge with MARV or EBOV [14,15]. This contrasts with the recall response observed in the VSV-SUDV28 group, in which our CIBERSORTx analysis identified no changes in innate immune clusters (like monocytes), but rather the development of a memory response that included increased memory B cell abundance. Another notable difference is the upregulation of genes needed for erythrocyte differentiation/hemopoiesis and heme metabolism in the VSV-SUDV7 group at 21–35DPC. A clinical hallmark of filovirus infection is severe hemorrhage leading to hemolysis, further tissue injury, edema, and reduced oxygen delivery to tissues [52,53]. Heme released during hemolysis is toxic and can cause oxidative damage to tissues [54]. Heme oxygenase 2 (HMOX2) is the enzyme responsible for breaking down heme [54] and was increased at 21–35DPC. *ABCB10,* a mitochondrial transporter protein that plays a crucial role in erythropoiesis and protecting erythrocytes from oxidative stress [55], was upregulated. Altogether, the upregulation of genes associated with these clinical features may indicate that while VSV-SUDV vaccination as late as 7 days before SUDV challenge is protective, there may be subclinical features of the infection that have not yet been appreciated.

The transcriptional responses observed in our VSV-SUDV group align with findings from previous investigations of VSV-vectored vaccines against other filoviruses. In vaccinated animals, we observed a controlled innate immune response coupled with the development of a rapid antibody responses, while unprotected animals showed large transcriptional alteration indicative of a cytokine storm and fatal disease mirroring findings with VSV-EBOV, VSV-MARV, and VSV-TAFV [14–16,21–23,56]. Similar to VSV-MARV and VSV-EBOV vaccination, interferon stimulated genes were upregulated as early as 3 days post vaccination [14,15,21]. Upregulation of complement factors post challenge was reported following VSV-EBOV vaccination, while previous studies of VSV-MARV have shown significantly higher ADCP and ADCD [23,57].

The rapid protection with VSV-SUDV vaccination administered as late as 7 days before challenge is consistent with other VSV-vectored vaccines, including VSV-EBOV and VSV-MARV, which demonstrate full protective immunity within 7 days and patrial immunity after 3 days of vaccination [14,15]. Interestingly, despite full protective immunity, we observed potential subclinical features of SVD in animals vaccinated only 7 days before challenge. A future study is needed to do a comprehensive investigation of how the various GP inserts modulate host responses to VSV vectored vaccines.

Our study is not without limitations, most importantly the low n = 3 per groups and the reliance on in silico methods of cell frequency estimation due to logistical complications in the ability to do flow cytometry. Our study revealed the transcriptional changes induced by VSV-SUDV vaccination. Additionally, while VSV-SUDV7 provides complete rapid protection from fatal disease, we show transcriptional evidence of subclinical features of infection. With the likelihood of continued SUDV outbreaks, future work is needed to determine the minimum interval time from vaccination to exposure for protection.

## Materials and methods

### Ethics statement

Animal work was approved by the Rocky Mountain Laboratories (RML) Animal Care and Use Committee (ACUC) and was performed per the description in [6] in accordance with the recommendations in the Guide for the Care and Use of Laboratory Animals of the National Institute of Health, the Office of Animal Welfare, and the United States Department of Agriculture. Endpoint criteria were specified by RML ACUC-approved parameters.

### Vaccines and challenge virus

Cynomolgus macaques were vaccinated intramuscularly (IM) with 1x10^7^ PFU of VSV-based vaccine vectors expressing the EBOV-Kikwit GP (VSV-EBOV) [58] or SUDV-Gulu GP (VSV-SUDV) [6] at -28 or -7 days post-challenge (DPC). All groups were challenged IM at DPC0 with 1x10^4^ TCID_50_ of SUDV-Gulu as described [6].

### Sample description

We used a subset of the samples described previously [12]. Samples from three animals randomly selected per group were used for transcriptomic analysis: 1) control group vaccinated with VSV-EBOV at DPC-28 (VSV-EBOV28), 2) group vaccinated with VSV-SUDV at DPC-28 (VSV-SUDV28), and 3) group vaccinated with VSV-SUDV at DPC-7 (VSV-SUDV7). Samples from the following time points were collected for analysis DPC-28, -21, 0, 3, and 5 for VSV-EBOV28, DPC-28, -21, 0, 3, 6, 21, and 35 for VSV-SUDV28, and at DPC-7, 0, 3, 6, 21, and 35 for VSV-SUDV7. Animals in the VSV-EBOV28 group reached endpoint criteria DPC5. These time points allowed us to capture days of vaccination, peak vaccine response, challenge, peak recall response and plateau for surviving animals and fatal disease for animals that succumbed. Our previous study included an additional control group vaccinated with VSV vectored Lassa virus (VSV-LASV) GP which displayed the same fatal outcome as the VSV-EBOV28 group. Given the known lack of cross protection provided by VSV-EBOV, we utilized the VSV-EBOV28 group as the sole control for our transcriptomic analysis to focus the study on filoviruses.

### Bulk RNA library preparation and analysis

RNA was extracted from whole blood samples and cDNA Libraries were generated as described [16] and sequenced on an illumina Novaseq X.

Gene expression analysis was performed using EdgeR [59], STEM [17], and MaSigPro [18] as described [16]. CIBERSORTx, an *in-silico* flow cytometry method, was utilized to estimate relative proportions of cell types from gene expression data in heterogeneous samples [19,20]. Gene counts normalized as transcripts per million (TPM) were used as input. We used the publicly available LM22 Signature Matrix as reference and performed the analysis with 500 permutations and batch mode for correction of bulk data. Comparisons of estimated immune cell frequencies were calculated with two-way ANOVA (Prism 10). Heatmaps, bar plots, bubble plots, and Venn diagrams were generated using R version 4·1 and ggplot2 package. Error bars indicate SEM.

## Supporting information

S1 Fig Transcriptional alterations associated with lethal disease. (A) Line graph of the average TPM values for each gene cluster post challenge for VSV-EBOV28. (B-C) Bar graph representing GO terms for DEGs. Color indicates log10(q.value). Length indicates the number of DEGs. (D-E) Heatmap of average TPM values of selected DEGs.
(EPS)

S2 Fig Changes in immune cell frequencies after SUDV challenge. Bubble plot of estimated cell population frequencies using CIBERSORTx with two-way ANOVA between challenge timepoints and groups. Size indicates the predicted frequencies of the indicated cell subset and color indicates –log10(p.value) of the indicated timepoint relative to 0 DPC for the group. # p value < 0·05 and ## p value <0·01 of the indicated timepoint in VSV-SUDV28 relative to VSV-EBOV28. * p value < 0·05 and ** p value <0·01 of the indicated timepoint in VSV-SUDV7 relative to VSV-SUDV28.
(EPS)

S1 Table Differential gene expression. (XLSX)
